# Spatial Stability of Functional Networks: A Measure to Assess the Robustness of Graph-Theoretical Metrics to Spatial Errors Related to Brain Parcellation

**DOI:** 10.3389/fnins.2021.736524

**Published:** 2022-02-18

**Authors:** Francesca Bottino, Martina Lucignani, Luca Pasquini, Michele Mastrogiovanni, Simone Gazzellini, Matteo Ritrovato, Daniela Longo, Lorenzo Figà-Talamanca, Maria Camilla Rossi Espagnet, Antonio Napolitano

**Affiliations:** ^1^Medical Physics Department, Bambino Gesù Children’s Hospital IRCCS, Rome, Italy; ^2^Neuroradiology Unit, NESMOS Department, Sant’Andrea Hospital, La Sapienza University, Rome, Italy; ^3^Neuroradiology Service, Department of Radiology, Memorial Sloan Kettering Cancer Center, New York, NY, United States; ^4^Neuroscience and Neurorehabilitation Department, Bambino Gesù Children’s Hospital – IRCCS, Rome, Italy; ^5^Health Technology and Safety Research Unit, Bambino Gesù Children’s Hospital – IRCCS, Rome, Italy; ^6^Neuroradiology Unit, Imaging Department, Bambino Gesù Children’s Hospital, IRCCS, Rome, Italy; ^7^NESMOS, Neuroradiology Department, S. Andrea Hospital Sapienza Rome University, Rome, Italy

**Keywords:** parcellation, graph-theoretical measures, functional, fMRI, brain connectivity, stability

## Abstract

There is growing interest in studying human brain connectivity and in modelling the brain functional structure as a network. Brain network creation requires parcellation of the cerebral cortex to define nodes. Parcellation might be affected by possible errors due to inter- and intra-subject variability as a consequence of brain structural and physiological characteristics and shape variations related to ageing and diseases, acquisition noise, and misregistration. These errors could induce a knock-on effect on network measure variability. The aim of this study was to investigate spatial stability, a measure of functional connectivity variations induced by parcellation errors. We simulated parcellation variability with random small spatial changes and evaluated its effects on twenty-seven graph-theoretical measures. The study included subjects from three public online datasets. Two brain parcellations were performed using FreeSurfer with geometric atlases. Starting from these, 100 new parcellations were created by increasing the area of 30% of parcels, reducing the area of neighbour parcels, with a rearrangement of vertices. fMRI data were filtered with linear regression, CompCor, and motion correction. Adjacency matrices were constructed with 0.1, 0.2, 0.3, and 0.4 thresholds. Differences in spatial stability between datasets, atlases, and threshold were evaluated. The higher spatial stability resulted for Characteristic-path-length, Density, Transitivity, and Closeness-centrality, and the lower spatial stability resulted for Bonacich and Katz. Multivariate analysis showed a significant effect of atlas, datasets, and thresholds. Katz and Bonacich centrality, which was subject to larger variations, can be considered an unconventional graph measure, poorly implemented in the clinical field and not yet investigated for reliability assessment. Spatial stability (SS) is affected by threshold, and it decreases with increasing threshold for several measures. Moreover, SS seems to depend on atlas choice and scanning parameters. Our study highlights the importance of paying close attention to possible parcellation-related spatial errors, which may affect the reliability of functional connectivity measures.

## Introduction

In the last few years, the interest in graph theory applications to functional connectivity (FC) data has grown exponentially. In neuroimaging, FC can be assessed on functional magnetic resonance imaging (fMRI) by measuring fluctuations of the blood oxygenation level-dependent (BOLD) signal in the brain (Ogawa et al., 1990). Brain regions with a similar function show correlated spontaneous oscillations at rest, so that resting-state time-series enable to infer the corresponding FC (Biswal et al., 1995; Lowe et al., 1998; Cordes et al., 2000; De Luca et al., 2005; Fox and Raichle, 2007). In functional networks, system elements (nodes) represent brain regions and relationships between them (edges) represent functional interactions. Once nodes are defined, the network structure can be estimated as a connection matrix, which can be computed as a correlation between the average time series of each node (Sporns et al., 2005). Network measures can be extracted from correlation matrices to describe the characteristics of individual regions. Each measure characterizes many different features providing a powerful means to classify and predict several brain disorders. The application of graph theory to clinical neuroscience studies shed light on the basic organization of the brain (Supekar et al., 2008; Smith et al., 2011; Li et al., 2020, 2021). Also, hypo- and hyper-connectivity may represent biomarkers for different diseases (Bassett et al., 2008; Kocevar et al., 2016; Li et al., 2019; Mazrooyisebdani et al., 2020; Xiang Y. et al., 2020; Chen et al., 2021).

A critical step in the construction of functional networks is node definition (Sporns, 2011; Eickhoff et al., 2015).

Most common nodal definition schemes are based on individual voxels and brain subdivisions into a set of distinct regions (parcellation). Voxel-level networks consider each voxel separately as a node and compute correlations between time-series of each voxel. Despite producing a network with a very high resolution (>10^4^ nodes) (van den Heuvel et al., 2008; Zalesky et al., 2011), this approach is computationally expensive, sensitive to noise, and difficult to interpret (Craddock et al., 2012; Thirion et al., 2014). The process of dividing the cerebral cortex and inner structure into structurally or functionally distinct regions is usually referred to as parcellation. From an initial application of graph theory to strictly voxel-based neuroimaging, more recent studies implemented parcellation for node definition in functional networks. Several parcellation methods have been proposed to define regions of interest (ROIs) for network analysis, with different resolution levels (number of parcels). The most common parcellation approaches are based on cytoarchitectonic or myeloarchitectonic information (Brodmann and Garey, 2006; Zilles and Amunts, 2010). Alternatively, “*a priori*” anatomical parcellation is often employed (Tzourio-Mazoyer et al., 2002; Desikan et al., 2006). However, such parcellation methods are usually generated on an individual basis, and brain atlases rely on the state of our knowledge of brain structure. Alternatively, random parcellation schemes can be used, including a variable number of regions usually in the order of 10^2^ to 10^3^ or more, but few studies demonstrated that such parcellation may produce loss of connectivity information (Smith et al., 2011). Recently, some studies used random parcellation as a null model to test a fixed parcellation approach (Arslan et al., 2018; Messé, 2020). Spherical ROIs centred on stereotaxic coordinates (Fair et al., 2009; Dosenbach et al., 2010) can also be used, but their application to structural models may be difficult. Numerous connectivity-driven parcellation methods from fMRI data have been introduced to define network nodes (Arslan et al., 2018), often in association with clustering techniques (Craddock et al., 2012; Thirion et al., 2014). In these approaches, brain regions are defined by connectivity patterns. Particularly, a voxel-wise map of BOLD signal temporal correlations is created, then voxels are clustered into groups to define region boundaries. These methods include k-means clustering (Tomassini et al., 2007; Golland et al., 2008; Mezer et al., 2009), hierarchical clustering (Bellec et al., 2010; Mumford et al., 2010; Moreno-Dominguez et al., 2014; Arslan and Rueckert, 2015), growing clustering (Bellec et al., 2006; Gilbody et al., 2007; Blumensath et al., 2013), spectral clustering (van den Heuvel et al., 2008; Craddock et al., 2012; Shen et al., 2013; Arslan et al., 2015, 2016; Parisot et al., 2016a), Markov random field technique (Ryali et al., 2013; Honnorat et al., 2015; Parisot et al., 2016b), Gaussian mixture models (Lashkari et al., 2010; Yeo et al., 2011), meta-analytic connectivity techniques (Eickhoff et al., 2011; Power et al., 2011), dictionary learning (Varoquaux et al., 2011), edge detection (Cohen et al., 2008; Laumann et al., 2015; Gordon et al., 2016), independent component analysis (ICA) (Beckmann and Smith, 2004; Smith et al., 2009), and Bayesian modelling (Baldassano et al., 2015), often with overlap in defined brain areas (Eickhoff et al., 2015). Other studies used a multimodal approach to parcellate the brain, implementing both anatomical and data-driven parcellation methods (Glasser et al., 2016) (see Arslan et al., 2018 for a review and comparison of existing parcellation methods) (Arslan et al., 2018). Recent studies have attempted to incorporate information about cross-subject variability into the atlas generation algorithms themselves (Kong et al., 2019). Despite all this evidence, a universally accepted parcellation method is still missing.

In connectivity studies, individual differences of cortical morphometry are sometimes dismissed as “noise” – perhaps reflecting measurement errors or non-significant variability (Chiarello et al., 2016). Caspers observed an interindividual variability of brain-area topography, related to interobserver variability of the definition of cytoarchitectonic borders based on visual inspection of histological sections, or interindividual variability of cytoarchitecture (Caspers et al., 2006). Some authors investigated intersubject variability of cortical anatomy, showing consistent hemispheric asymmetries (Kang et al., 2012), as well as differences in sulcal and gyral anatomy (Caulo et al., 2007). Other authors reported regional differences of cortical morphometry across individuals, demonstrating an association between the extent of the region’s between-subject variability and structural asymmetry (Chiarello et al., 2016). During parcellation, true anatomical borders are only marginally approximated because these are often subjective (Fornito et al., 2012). Kennedy computed the volume of 48 parcels (for each hemisphere) in twenty subjects and assessed the variation in size of volume of individual parcels among the twenty brains, finding a coefficient of variation range from 11.1% (insula) to 49.0% (occipital pole) (Kennedy et al., 1998). Recent studies showed that gyral definitions were not identical across parcellation types, with differences in bordering landmarks that could very likely be misinterpreted, leading to a knock-on effect on associated structures, ROI volumes, and corresponding morphometrics (Mikhael et al., 2018; Mikhael and Pernet, 2019). Also, acquisition noise and misregistration may compromise the location of regional boundaries between subjects and different scans of the same subject (de Reus and van den Heuvel, 2013), possibly distorting the areas of interest and leading to poor parcellation mapping. Crucial aspects to be considered when the brain is parcellated include the effect of parcellation resolution (i.e., the number of regions in a template), parcellation type (Sala-Llonch et al., 2019), parcellation template, and granularity (Bassett et al., 2008; Wang et al., 2010; de Reus and van den Heuvel, 2013).

Discrepancies in brain boundaries could induce connectivity map variations. Parcellation errors and poor node definition at the single-subject level affect the connectivity analysis, distorting the estimation of network interactions (Smith et al., 2011; Arslan et al., 2015). Since graph theoretic measures are affected by the selected parcellation (Wang et al., 2010; Arslan et al., 2018), errors in node definition for brain network analysis could produce misleading results and erroneous interpretations of cognitive processes in healthy subjects or patients (Stanley et al., 2013), e.g., producing false positive (and false negative) and significant differences in the topological organization of brain functional networks. Caution should be exercised when evaluating functional connectivity changes in longitudinal studies due to result variability (Klobušiakova et al., 2019; Oldehinkel et al., 2019).

We introduced spatial stability (SS), as a definition of parcellation change effects on functional connectivity variations. With SS analysis, we investigated network resilience to small parcellation variations and the reliability of functional connectivity results. To do this, we simulated parcellation variability reproducing random small spatial changes and then we evaluated the effects of these parcellations on graph-theoretical measures.

Our aim was to evaluate SS of graph-theoretical measures and to identify which of them are more reproducible when brain parcellation is affected by spatial errors. In this work, we do not investigate FC itself, but only subsequently derived graph measures.

## Materials and Methods

### Dataset

The study included healthy subjects from three public online datasets: COBRE dataset^1^, Olin Neuropsychiatric Research Center ABIDEII dataset (ONRC), and Indiana University ABIDEII dataset (IU)^2^. All of these include both functional and anatomical MR data. Few subjects were excluded due to poor image contrast after T1w visual quality control^3^. Particularly, 3 subjects were excluded from the ONRC ABIDEII original dataset, 3 subjects were excluded from the INDIANA ABIDEII original dataset, and 9 subjects were excluded from the COBRE original dataset due to poor image contrast. As a result, we selected 61 subjects (COBRE) from COBRE, 32 subjects from ONRC ABIDEII, and 17 subjects (IU) from INDIANA UNIVERSITY ABIDEII. All the data were acquired on 3T scanners (ONRC: Siemens Skyra; IU: Siemens TrioTim; COBRE: Siemens TrioTim). A brief summary of the demographic data included in the datasets is shown in Table 1. Anatomical and Rs-fMRI acquisition parameters are given in Tables 2, 3. Datasets were explicitly waived IBR approval due to publicly available fully anonymized data.

**TABLE 1:** Summary of the demographic data included in the datasets.

Datasets	Groups	*n*	Age	Sex (M/F)
COBRE	Healty subjects	61	18–65	43/18
ONRC	Healty subjects	32	19–30	18/14
IU	Healty subjects	17	19–37	12/5

**TABLE 2:** Anatomical acquisition parameters of datasets.

MPRAGE	COBRE	ONRC	IU
Magnetic field strength (T)	3	3	3
TR (ms)	2,530	2,200	2,400
TE (ms)	1.64	2.88	2.3
T1 (ms)	900	794	1,000
Averages	1	1	1
Pixel bandwidth (Hz/Px)	650	200	210
Acquisition matrix	256 × 256 × 192	208 × 320 × 220	320 × 320 × 256
Flip angle	7	13	8
FOV (mm)	256 × 256	256 × 256	224 × 224
Slice thickness (mm)	1	0.8	0.7
Total scan time (min)	6	3	7

**TABLE 3:** Rs-fMRI acquisition parameters of datasets.

Rs – fMRI	COBRE	ONRC	IU
Magnetic field strength (T)	3	3	3
Scanning sequence	EPI	EPI	EPI
TR (ms)	2,000	475	813
TE (ms)	29	30	28
Slice thickness (mm)	3.5	3	3.4
Pixel bandwidth (Hz/Px)	2170	2,604	2,604
Number of slices	33	48	42
Number of volumes	150	947	433
Acquisition matrix	64 × 64	80 × 80	64 × 64

### Data Preprocessing

MPRAGE sequences were preprocessed using the FreeSurfer 5.4 pipeline (Fischl, 2012). In particular, cortical and subcortical segmentation processing included motion correction (Reuter et al., 2010), skull stripping (Ségonne et al., 2004), extraction of the cortical surface (Fischl et al., 2002; Ségonne et al., 2004), and spatial normalization onto the FreeSurfer surface template (FsAverage). A smooth, continuous, two-dimensional brain surface was then obtained based on high-resolution MPRAGE images. We used the QA tools^4^ for the processing of the structural data on FreeSurfer and for quality control of the segmentations.

### Parcellation

Two atlases were used in this study for parcellating each subject’s brain: standard atlasDKT40 and Destrieux atlas. These two atlases parcellate the FsAverage template into 64 anatomical regions of interest (64 Standard Parcels: SP) and 150 anatomical regions of interest (150 SP), respectively. Starting from the 2 aforementioned atlases, 100 new template-based atlases were randomly generated for each subject using the algorithm described below. Each of the newly generated atlases contained the same number of modified parcels (MP) as the original reference. Each of them was obtained by randomly increasing the area of a fixed number of SP and reducing the area of neighbour parcels with a rearrangement of vertices. The SP and MP obtained for each subject on the FsAverage surface were registered on individual surfaces. Once surface parcels were generated, these were converted in nifty volume files using FreeSurfer.

### Algorithm

A random modification algorithm was implemented on python to simulate 100 new atlases starting from the original references (standard atlasDKT40 and Destrieux atlas). Particularly, the algorithm implemented SP on each subject included in the pipeline. To do this, the parcellation template is resampled on subject surfaces. Then, 100 MP were randomly generated for each subject. Algorithm inputs are (i) the standard atlas to modify, (ii) the percentage of total brain surface vertices to move from the parcels (T%), (iii) the number of parcels to change (N), (iv) the number of random modified parcellation to create for each subject (m), and (v) subject data.

Algorithm outputs are (i) SP for each subject and (ii) m MP for each subject.

The algorithm works on FreeSurfer’s inflated brain surfaces. Parcellation atlases on these surfaces consist of a given labelling for each vertex referring to the parcel to which it is included. Each vertex in the brain surface belongs to the brain’s mesh. A mesh face consists of three non-collinear vertices. When the brain surface is parcelled, a face where all three vertices have the same label is considered as an “internal face” included in a parcel, whereas a face with at least one label different from the others is considered a “boundary face” included in two (or three) parcels. Finally, the set of faces of a parcel does not contain holes.

**FIGURE 1:**
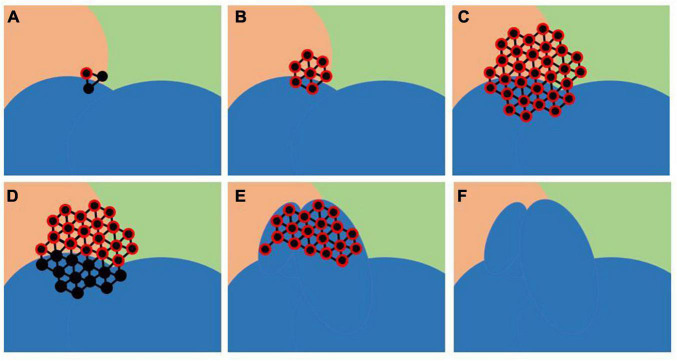
Modified Parcels (MP) creation: SP to modify (blue) and a vertex external to this selected SP, belonging to its boundary face, were randomly chosen, and a growing set of vertices G that initially contains only this chosen vertex is defined **(A)**. Vertices adjacent to each vertex in G are added to G iteratively **(B,C)**. Vertices belonging to the SP were removed from G **(D)**. Resulted G was moved in SP resulting in MP **(E,F)**.

Given the number of parcels N in order to modify the total percentage of vertices T%, the following steps are performed for each randomly selected parcel i from N:

(1) . Selection of a set of vertices G_i by taking three random vertices that belong to boundary faces of a randomly selected SPi in blue shown in Figure 1 (Figure 1A).
(2) . Expanding G_i by adding new vertices adjacent to a vertex (red encircled in Figure 1A) in G_i included in the parcel nearby to SPi (Figure 1B).
(3) . Expanding G_i by adding new vertices adjacent to each vertex in G_i (Figure 1C).
(4) . Removing all vertices in G_i that belong to the chosen SPi (Figure 1D).
(5) . Repetition of step 2 until the total number of vertices in the growing set G_i is higher than the fixed value e_i defined as

$$e{\_}_{i}=\frac{T_{\%}\,V_{i}\,V_{\mathrm{tot}}}{100\,\sum_{j=1}^{N}V_{j}},$$

whereby Vi is the total number of vertices in the selected SPi, Vj is the total number of each brain parcel, and Vtot is the total number of vertices in the brain mesh.
(6) . MP generation: all vertices in G_i are labelled to be included in the SPI (Figures 1E,F). Particularly, considering SPi and a neighbouring parcel, the labels of vertices in G_i are changed from the label of the neighbouring parcel to the label of the SPi to reduce the size of the first and increase the size of the other.
(7) . This iterative process is repeated for each random selected SPi.

**FIGURE 2:**
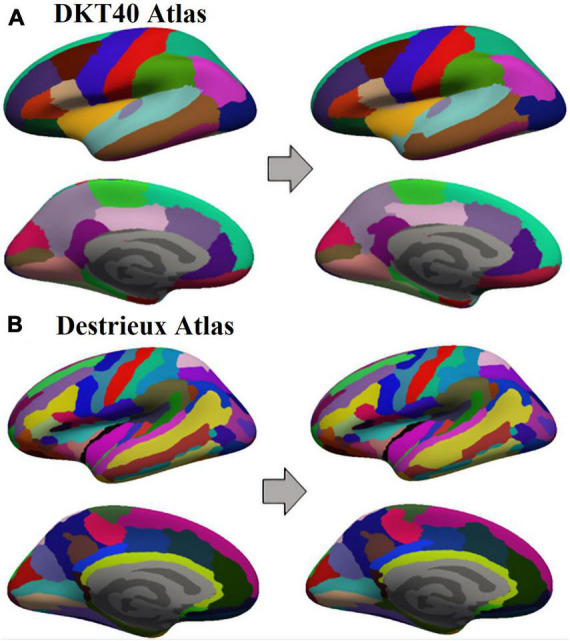
Example of Standard Parcels (SP) and Modified Parcels (MP) obtained on an inflated subject surface in one of its 100 random iterations, starting from DKTatlas40 **(A)** and Destrieux atlas **(B)**.

We reported an example of SP and MP obtained on an inflated subject surface in one of its 100 random iterations, shown in Figure 2. In short, we have chosen the number of vertices to add to the size of a parcel so that, for example, if a randomly selected parcel to be expanded in size was twice the size of another randomly selected parcel to be expanded, we would add twice the number of vertices e_i to the first parcel than to the second.

We performed spatial variations of brain parcels to reproduce a mean coefficient of variation of individual parcel volume equal to 10%, reflecting the minimum coefficient of between-subject variability for a parcellation unit (Kennedy et al., 1998). Particularly, in order to reproduce a coefficient of variation equal to 10%, we chose N equal to 30% of SP and T% equal to 3%.

### Brain Connectivity Analysis

A BOLD visual quality control of functional MRI data was performed (see text footnote 3). All fMRI data were filtered with linear regression to reduce the effects of low-frequency drift. High-frequency physiological noise was extracted with the CompCor method (Behzadi et al., 2007), and motion correction was applied using the mcflirt fsl command^5^. The first volume of fMRI data was used to realign and register fMRI data to the subject’s structural image. One hundred brain networks were developed for each analysis: the mean time series from each parcel were extracted, and connectivity matrices were created computing Pearson’s correlation coefficient between parcel average time series as a measure of the functional connectivity between pairs of regions. As a result, 100 weighted and unweighted adjacency matrices were constructed per subject with 0.1, 0.2, 0.3, and 0.4 thresholds.

### Computation of Graph Measures

In order to assess the properties of each node and the whole network organization, 27 indirect network measures were computed in python using btc^6^ and NetworkX^7^ libraries, including centrality, clustering, core, degree, distance, modularity, physical connectivity, and similarity measure classes. Weighted and binary measures were derived starting from the respective adjacency matrices. Weighted measures were computed when the algorithm required a weighted adjacency matrix or allowed to choose from weighted or binary adjacency matrices as input. Binary measures were employed when no weighted counterpart was available (i.e., subgraph centrality, flow coefficient, and k-coreness). A summary of the measures implemented in this study is displayed in Table 4. The evaluated metrics included global and local measures. Global measures are Assortativity, Characteristic path length, Community Louvain, Density, Global Efficiency, Modularity Louvain, Modularity Finetune, and Transitivity. Details about the graph measures are reported in Supplementary Materials.

**TABLE 4:** Graph theoretical measures included in the study.

Weighted measures	Binary measures
***Centrality measures*** Bonacich centrality Betweenness centrality Eigen vector centrality Shortcuts centrality Pagerank centrality Closeness centrality Katz centrality Communicability betweenness centrality	***Centrality measures*** Subgraph centrality Flow coefficient k-coreness centrality
***Clustering measures*** Get components Clustering coefficient Transitivity	
***Core measures*** Assortativity Core periphery	
***Degree measures*** Degree Strength	
***Distance measures*** Global efficiency Local efficiency Characteristic path length	
***Modularity measures*** Modularity finetune Modularity louvain Community louvain	
***Physical connectivity measures*** Density	
***Similarity measures*** Topological overlap Matching index	

### Variation Factor

To evaluate the SS of graph metrics, we introduced the brain connectivity variation factor (VF) over all simulated parcel variations: a measure of reproducibility of the functional connectivity analysis results in terms of graph measures. VF is proportional to the difference between metric values derived by SP and metric values derived by MP parcellations. Therefore, a graph metric with a higher VF will be less stable in terms of SS, being more vulnerable to parcellation-related spatial errors. Conversely, a graph measure with a lower VF value will be more reproducible when the brain is affected by spatial parcellation errors.

For local measures, VF was defined as


$$\text{VF}\left(\%\right)=\frac{1}{\text{N}}\frac{1}{\text{P}}\sum_{p=1}^{P}\sum_{i=1}^{N}\frac{\sqrt{{\left({\text{x}}_{\mathrm{ip}}^{\mathrm{MP}}-{\text{x}}_{\mathrm{ip}}^{S\,P}\right)}^{2}}}{\sqrt{{\left(\frac{x_{\mathrm{ip}}^{\mathrm{MP}}+x_{\mathrm{ip}}^{\mathrm{SP}}}{2}\right)}^{2}}}100$$

whereby $x_{i\,p}^{M\,P}$ and $x_{i\,p}^{S\,P}$ correspond to the value of the x graph measure under investigation in subject i, for parcel p, for modified and standard parcellations, respectively; N and P represent the total number of subjects and parcels, respectively.

For global measures, the brain connectivity VF was defined as


$$\text{V}F\left(\%\right)=\frac{1}{\text{N}}\sum_{i=1}^{N}\frac{\sqrt{{\left({\text{x}}_{i}^{M\,P}-{\text{x}}_{i}^{S\,P}\right)}^{2}}}{\sqrt{{\left(\frac{x_{i}^{M\,P}+x_{i}^{S\,P}}{2}\right)}^{2}}}100$$

whereby $x_{i}^{M\,P}$ and $x_{i}^{S\,P}$ correspond to the value of the measure under investigation in subject i, for modified and standard parcellations, respectively; N is the total number of subjects.

Variation factor was computed for each of the 100 randomly modified parcellation instance and for each analysed measure. Differences in VF between datasets, atlases, and thresholds were also evaluated.

The pipeline implemented in this study, as described above, is implemented on python and is publicly available at https://github.com/mri-group-opbg/effect-of-parcellation-changes-analysis.

### Statistical Analysis

Statistical analysis was carried out with SPSS software (PAWS Statistics 18.0). Statistical significance was set at 0.05. To test the effect of dataset (ONRC, IU and COBRE), atlas (DKTatlas40 and aparc2009s), and threshold (0.1, 0.2, 0.3, 0.4) on VF values for each measure, we run a MANOVA analysis, whereby the VF values were considered as variables and the dataset, atlas, and threshold served as fixed factors. The analysis was performed with Pillai’s trace statistic. To explore significant results derived from multiple comparisons, we included a *post hoc* analysis with Bonferroni correction for dataset and threshold.

## Results

The MANOVA analysis performed with Pillai’s trace showed significant atlas effect (DKTatlas40 vs. aparc2009s) (*F*(27,847) = 213.680; *p* < 0.001), dataset effect (ONRC vs. IU vs. COBRE) *F*(54,1696) = 6.193; *p* < 0.001), and threshold effect (0.1 vs. 0.2 vs. 0.3 vs. 0.4) (*F*(81,2547) = 30.283; *p* < 0.001).

**FIGURE 3:**
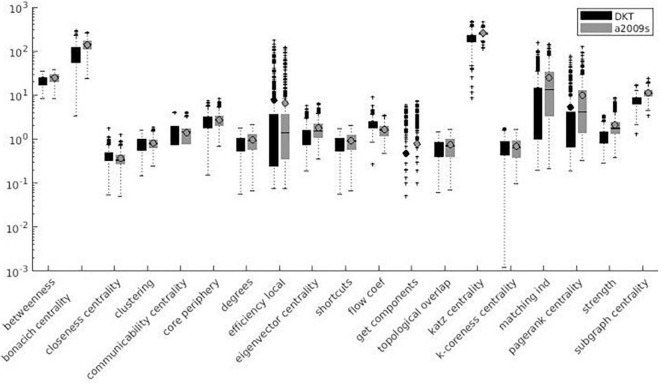
Local measures variation factor grouped with atlases.

**FIGURE 4:**
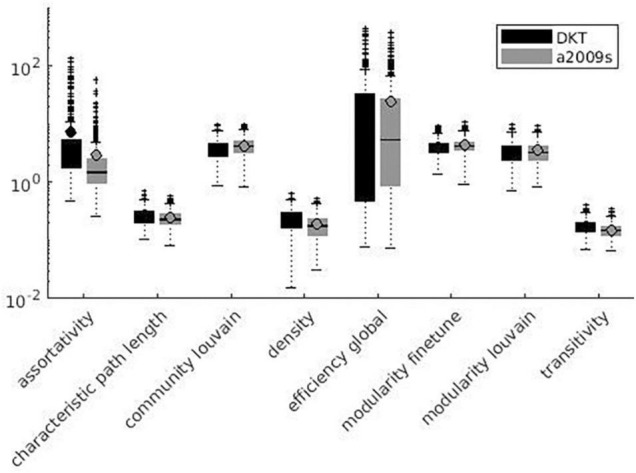
Global measures variation factor grouped with atlases.

**FIGURE 5:**
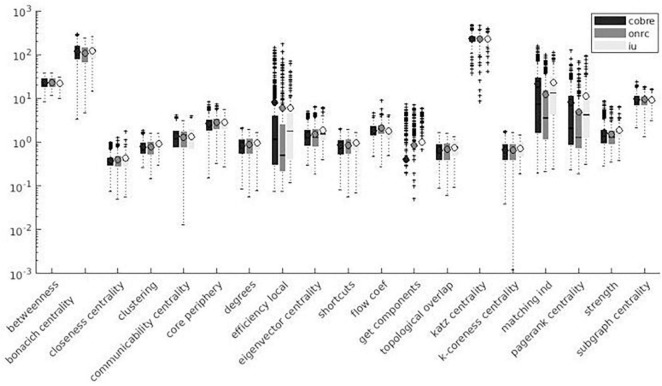
Local measures variation factor grouped with datasets.

Variation factor values were lower than 1% for characteristic path length, density, transitivity (outliers included), and closeness centrality (outliers excluded). VF values were lower than 10%, including outliers, for community Louvain, Modularity Finetune and Modularity Louvain, closeness centrality, communicability centrality, Eigen-vector, Shortcuts, Flow coefficient, Get components, K-coreness, degree, strength, Clustering Coefficient, core periphery, and Topological overlap and excluding outliers for Assortativity and local Efficiency. VF values ranged from 10 to 100% for Betweenness centrality, global Efficiency (excluding outliers), Matching index, Pagerank centrality (excluding outliers), and Subgraph centrality. VF values were higher than 100% for Katz and Bonacich centrality. Figures 3–5 summarize the above observations.

### Effect of Atlas

Variation factor results were affected by the atlas choice for 20 measures. Table 5 shows mean VF values for every measure with atlas grouping and resulting significant differences in VF. VF was higher for Destrieux atlas than DKT atlas for all significant metrics except for Assortativity, Characteristic path length, Closeness centrality, Density, Flow coefficient, and Transitivity. Figures 3, 4 show results grouped by atlas for local and global measures.

**TABLE 5:** VF values for measure with atlas grouping and significant differences in VF.

Measure	Group	Mean (dev)	F (pval)
Assortativity	DKT	7.11 (0.57)	*F*(1,873) = 32.31 (10^–8^)
	a2009s	2.83 (0.57)	
Betweenness centrality	DKT	21.36 (5.52)	*F*(1,873) = 62.11 (10^–15^)
	a2009s	24.44 (5.52)	
Bonacich centrality	DKT	92.62 (2.28)	*F*(1,873) = 233.92 (10^–47^)
	a2009s	138.68 (2.28)	
Characteristic path length	DKT	0.27 (0.004)	*F*(1,873) = 26.56 (10^–7^)
	a2009s	0.25 (0.004)	
Closeness	DKT	0.43 (0.006)	*F*(1,873) = 32.59 (10^–8^)
centrality	a2009s	0.38 (0.006)	
Clustering coefficient	DKT	0.81 (0.01)	*F*(1,873) = 5.74 (10^–2^)
	a2009s	0.85 (0.01)	
Communicability betweenness centrality	DKT	1.32 (0.03)	NS
	a2009s	1.36 (0.03)	
Community louvain	DKT	3.81 (0.08)	*F*(1,873) = 23.81 (10^–6^)
	a2009s	4.32 (0.08)	
Core periphery	DKT	2.68 (0.06)	NS
	a2009s	2.83 (0.06)	
Degree	DKT	0.83 (0.01)	*F*(1,873) = 78.72 (10^–16^)
	a2009s	0.97 (0.01)	
Density	DKT	0.24 (0.003)	*F*(1,1873) = 100.41 (10^–22^)
	a2009s	0.20 (0.003)	
Efficiency_global	DKT	25.68 (2.35)	NS
	a2009s	23.05 (2.35)	
Efficiency_local	DKT	7.13 (0.94)	NS
	a2009s	6.06 (0.94)	
Eigenvector centrality	DKT	1.40 (0.04)	NS
	a2009s	1.84 (0.04)	
Shortcuts averagerange	DKT	0.81 (0.01)	*F*(1,873) = 87.60 (10^–20^)
	a2009s	0.96 (0.01)	
Flow coefficient	DKT	2.22 (0.03)	*F*(1,873) = 236.09 (10^–47^)
	a2009s	1.63 (0.03)	
Get components	DKT	0.60 (0.06)	*F*(1,873) = 15.759 (10^–5^)
	a2009s	0.90 (0.06)	
Topological overlap	DKT	0.65 (0.01)	*F*(1,873) = 53.19 (10^–13^)
	a2009s	0.75 (0.01)	
Katz centrality	DKT	200.45 (2.81)	*F*(1,873) = 217.62 (10^–44^)
	a2009s	255.09 (2.81)	
k-coreness centrality	DKT	0.69 (0.01)	NS
	a2009s	0.68 (0.01)	
Matching index	DKT	12.83 (1.16)	*F*(1,873) = 59.29 (10^–14^)
	a2009s	24.62 (1.16)	
Modularity finetune	DKT	4.05 (0.07)	*F*(1,873) = 17.62 (10^–5^)
	a2009s	4.46 (0.07)	
Modularity Louvain	DKT	3.59 (0.08)	NS
	a2009s	3.61 (0.08)	
Pagerank centrality	DKT	5.76 (0.61)	*F*(1,873) = 31.09 (10^–8^)
	a2009s	10.27 (0.61)	
Strength	DKT	1.25 (0.05)	*F*(1,873) = 166.83 (10^–35^)
	a2009s	2.12 (0.05)	
Subgraph centrality	DKT	7.45 (0.12)	*F*(1,873) = 569.19 (10^–97^)
	a2009s	11.27 (0.12)	
Transitivity	DKT	0.18 (0.002)	*F*(1,873) = 69.87 (10^–16^)
	a2009s	0.15 (0.002)	

### Effect of Dataset

**FIGURE 6:**
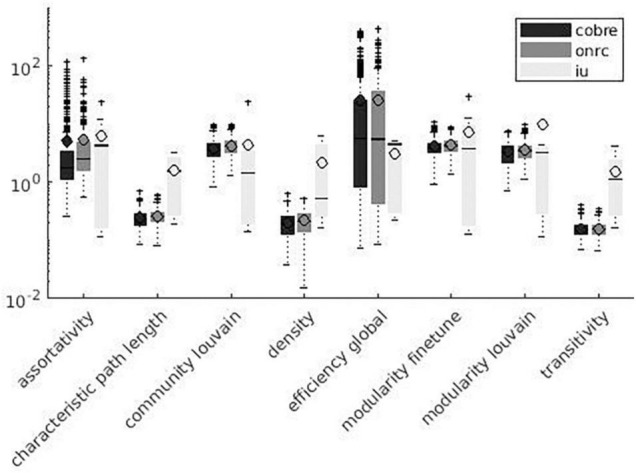
Global measures variation factor grouped with datasets.

A significant effect of the dataset on VF values was detected for 19 measures. Among significant measures, 15/19 metrics showed lower VF for the COBRE dataset and 13/19 metrics showed higher VF for the IU dataset. Table 6 shows dataset effects resulting from significant multiple comparisons and *post hoc* analysis. Figures 5, 6 show results grouped by dataset.

**TABLE 6:** VF values for measure with datasets grouping and significant differences in VF.

Measure	Group	Mean (dev)	F (pval)	*Post hoc* analysis significant results (pval)
Assortativity	COBRE	5.10 (0.51)	NS	
	ONRC	5.50 (0.70)		
	IU	4.29 (0.96)		
Betweenness centrality	COBRE	23.30 (0.26)	NS	
	ONRC	23.31 (0.36)		
	IU	22.09 (0.50)		
Bonacich centrality	COBRE	118.94 (2.02)	*F*(2,873) = 36.73 (10^–3^)	ONRC < COBRE (10^–3^)
	ONRC	107.25 (2.80)		ONRC < IU (10^–2^)
	IU	120.75 (2.02)		
Characteristic path length	COBRE	0.24 (0.003)	*F*(2,873) = 11.16 (10^–5^)	COBRE < ONRC (10^–3^)
	ONRC	0.26 (0.005)		COBRE < IU (10^–4^)
	IU	0.27 (0.006)		
Closeness centrality	COBRE	0.38 (0.005)	*F*(2,1029) = 11.13 (10^–5^)	COBRE < ONRC (10^–2^)
	ONRC	0.40 (0.007)		COBRE < IU (10^–5^)
	IU	0.43 (0.01)		ONRC < IU (10^–2^)
Clustering coefficient	COBRE	0.78 (0.01)	*F*(2,1029) = 20.00 (10^–9^)	COBRE < IU (10^–8^)
	ONRC	0.77 (0.01)		ONRC < IU (10^–8^)
	IU	0.94 (0.02)		
Communicability betweenness centrality	COBRE	1.39 (0.02)	NS	
	ONRC	1.31 (0.04)		
	IU	1.32 (0.05)		
Community louvain	COBRE	3.81 (0.07)	*F*(2,1029) = 7.70 (10^–4^)	COBRE < ONRC (10^–4^)
	ONRC	4.27 (0.09)		
	IU	4.11 (0.13)		
Core periphery	COBRE	2.60 (0.053)	*F*(2,1029) = 4.29 (10^–2^)	COBRE < ONRC (10^–2^)
	ONRC	2.84 (0.07)		
	IU	2.82 (0.10)		
Degree	COBRE	0.86 (0.01)	*F*(2,873) = 9.37 (10^–5^)	COBRE < IU (10^–5^)
	ONRC	0.86 (0.02)		ONRC < IU (10^–4^)
	IU	0.97 (0.02)		
Density	COBRE	0.20 (0.003)	*F*(2,873) = 16.40 (10–7)	COBRE < ONRC (10^–4^)
	ONRC	0.22 (0.004)		COBRE < IU (10^–7^)
	IU	0.23 (0.005)		
Efficiency global	COBRE	25.95 (2.076)	NS	
	ONRC	26.32 (2.87)		
	IU	20.83 (3.93)		
Efficiency local	COBRE	7.93 (0.83)	NS	
	ONRC	5.93 (1.15)		
	IU	5.93 (1.58)		
Eigenvector centrality	COBRE	1.45 (0.032)	*F*(2,873) = 18.22 (10^–8^)	COBRE < IU (10^–9^)
	ONRC	1.53 (0.044)		ONRC < IU (10^–5^)
	IU	1.86 (0.060)		
Shortcuts averagerange	COBRE	0.84 (0.01)	*F*(2,873) = 10.51 (10^–5^)	COBRE < IU (10^–5^)
	ONRC	0.86 (0.02)		ONRC < IU (10^–3^)
	IU	0.95 (0.02)		
Flow coefficient	COBRE	1.89 (0.03)	*F*(2,873) = 13.70 (10^–6^)	COBRE < ONRC (10^–5^)
	ONRC	2.08 (0.03)		IU < ONRC (10^–6^)
	IU	1.80 (0.05)		
Get components	COBRE	0.39 (0.05)	*F*(2,873) = 22.02 (10^–10^)	COBRE < ONRC (10^–7^)
	ONRC	0.85 (0.07)		COBRE < IU (10^–7^)
	IU	0.98 (0.09)		
Topological overlap	COBRE	0.66 (0.01)	*F*(2,873) = 11.27 (10^–5^)	COBRE < IU (10^–6^)
	ONRC	0.68 (0.01)		IU < ONRC (10^–4^)
	IU	0.76 (0.02)		
Katz centrality	COBRE	228.77 (2.49)	NS	
	ONRC	223.68 (3.43)		
	IU	230.84 (4.71)		
k-coreness centrality	COBRE	0.67 (0.01)	NS	
	ONRC	0.67 (0.01)		
	IU	0.72 (0.02)		
Matching index	COBRE	21.40 (1.03)	*F*(2,873) = 16.22 (10^–7^)	COBRE > ONRC (10^–7^)
	ONRC	12.12 (1.42)		ONRC < IU (10^–5^)
	IU	22.66 (1.95)		
Modularity finetune	COBRE	4.14 (0.07)	*F*(2,873) = 3.44 (0.03)	COBRE < ONRC (10^–2^)
	ONRC	4.43 (0.09)		
	IU	4.20 (0.13)		
Modularity louvain	COBRE	3.33 (0.07)	*F*(2,873) = 7.50 (10^–4^)	COBRE < ONRC (10^–2^)
	ONRC	3.62 (0.095)		COBRE < IU (10^–3^)
	IU	3.85 (0.13)		
Pagerank centrality	COBRE	8.06 (0.54)	*F*(2,873) = 12.67 (10^–6^)	ONRC < COBRE (10^–3^)
	ONRC	4.89 (0.75)		COBRE < IU (10^–2^)
	IU	11.09 (1.03)		ONRC < IU (10^–6^)
Strength	COBRE	1.66 (0.04)	*F*(2,873) = 6.81 (10^–3^)	COBRE < IU (10^–2^)
	ONRC	1.50 (0.06)		ONRC < IU (10^–4^)
	IU	1.89 (0.086)		
Subgraph centrality	COBRE	9.46 (0.11)	NS	
	ONRC	9.35 (0.15)		
	IU	9.28 (0.20)		
Transitivity	COBRE	0.16 (0.002)	*F*(2,873) = 12.78 (10^–6^)	COBRE < IU (10^–6^)
	ONRC	0.16 (0.002)		ONRC < IU (10^–5^)
	IU	0.18 (0.003)		

### Effect of Threshold

**FIGURE 7:**
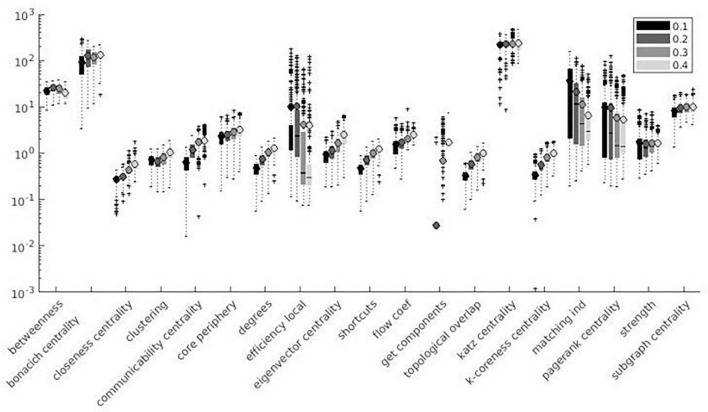
Local measures variation factor grouped with thresholds.

**FIGURE 8:**
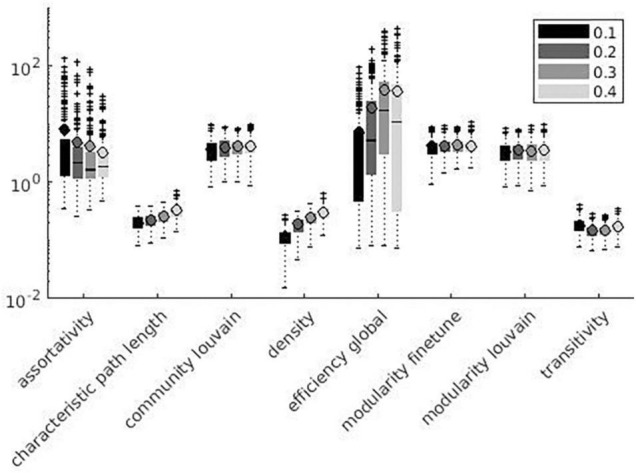
Global measures variation factor grouped with thresholds.

Variation factor was affected by threshold for 24 measures. Table 7 shows the test of between-subject effects and *post hoc* results for measures with significant differences in VF at different thresholds. VF increased with the threshold for all significant metrics, with the exception of Assortativity, Betweenness centrality, local Efficiency, Matching index, Pagerank centrality, and Transitivity (Table 7). These results are shown in Figures 7, 8.

**TABLE 7:** VF values for measure with thresholds grouping and significant differences in VF.

Measure	Group	Mean (dev)	F (pval)	*Post hoc* analysis significant results (pval)
Assortativity	0.1	7.94 (0.78)	*F*(3,873) = 7.74 (10^–5^)	0.1 > 0.2 (10^–2^)
	0.2	4.71 (0.78)		0.1 > 0.3 (10^–3^)
	0.3	4.13 (0.79)		0.1 > 0.4 (10^–5^)
	0.4	3.07 (0.79)		
Betweenness centrality	0.1	21.92 (0.40)	*F*(3,873) = 40.98 (10^–25^)	0.1 < 0.2 (10^–9^)
	0.2	25.37 (0.40)		0.1 < 0.3 (10^–5^)
	0.3	24.42 (0.40)		0.1 > 0.4 (10^–3^)
	0.4	19.88 (0.40)		0.2 > 0.4 (10^–21^); 0.3 > 0.4 (10^–15^)
Bonacich centrality	0.1	90.90 (3.12)	*F*(3,873) = 35.23 (10^–21^)	0.1 < 0.2 (10^–14^)
	0.2	124.80 (3.12)		0.1 < 0.3 (10^–7^)
	0.3	115.04 (3.12)		0.1 < 0.4 (10^–20^)
	0.4	131.84 (3.12)		0.3 < 0.4 (10^–4^)
Characteristic path length	0.1	0.21 (0.005)	*F*(3,873) = 105.64 (10^–58^)	0.1 < 0.2 (10^–2^); 0.1 < 0.3 (10–13);
	0.2	0.23 (0.005)		0.1 < 0.4 (10–52); 0.2 < 0.3 (10^–6^);
	0.3	0.27 (0.005)		0.2 < 0.4 (10–39); 0.3 < 0.4 (10^–17^)
	0.4	0.33 (0.005)		
Closeness centrality	0.1	0.28 (0.008)	*F*(3,873) = 321.21 (10^–140^)	0.1 < 0.2 (10^–3^)
	0.2	0.32 (0.01)		0.1 < 0.3 (10^–40^)
	0.3	0.44 (0.01)		0.1 < 0.4 (10^–124^); 0.2 < 0.3 (10^–23^)
	0.4	0.59 (0.01)		0.2 < 0.4 (19^–101^) 0.3 < 0.4 (10^–40^)
Clustering coefficient	0.1	0.74 (0.02)	*F*(3,873) = 81.05 (10–46)	0.1 < 0.3 (10^–4^)
	0.2	0.70 (0.02)		0.1 < 0.4 (10^–33^);
	0.3	0.84 (0.02)		0.2 < 0.3 (10^–7^)
	0.4	1.06 (0.02)		0.2 < 0.4 (10^–40^); 0.3 < 0.4 (10^–17^)
Communicability betweenness centrality	0.1	0.62 (0.04)	*F*(3,873) = 200.11 (10–99)	0.1 < 0.2 (10^–20^)
	0.2	1.17 (0.04)		0.1 < 0.3 (10^–67^)
	0.3	1.70 (0.04)		0.1 < 0.4 (10^–86^); 0.2 < 0.3 (10^–19^)
	0.4	1.88 (0.04)		0.2 < 0.4 (10^–32^); 0.3 < 0.4 (10^–3^)
Community louvain	0.1	3.70 (0.11)	*F*(3,873) = 7.06 (10–4)	0.1 < 0.3 (10^–3^)
	0.2	4.03 (0.11)		0.1 < 0.4 (10^–5^)
	0.3	4.19 (0.11)		0.3 < 0.4 (10^–5^)
	0.4	4.34 (0.11)		
Core periphery	0.1	2.33 (0.08)	*F*(3,873) = 25.52 (10–16)	0.1 < 0.3 (10^–7^)
	0.2	2.53 (0.08)		0.1 < 0.4 (10^–14^)
	0.3	2.96 (0.08)		0.2 < 0.3 (10^–3^)
	0.4	3.22 (0.08)		0.2 < 0.4 (10^–9^)
Degree	0.1	0.48 (0.02)	*F*(3,873) = 409.83 (10–166)	0.1 < 0.3 (10^–7^)
	0.2	0.76 (0.02)		0.1 < 0.4 (10^–14^)
	0.3	1.05 (0.02)		0.2 < 0.3 (10^–3^)
	0.4	1.30 (0.02)		0.2 < 0.4 (10^–9^)
Density	0.1	0.12 (0.004)	*F*(3,873) = 23.40 (10–14)	0.1 < 0.2 (10^–28^)
	0.2	0.19 (0.004)		0.1 < 0.3 (10^–75^)
	0.3	0.25 (0.004)		0.1 < 0.4 (10^–135^); 0.2 < 0.3 (10^–17^)
	0.4	0.30 (0.004)		0.2 < 0.4 (10^–63^); 0.3 < 0.4 (10–19)
Efficiency global	0.1	6.21 (3.21)	*F*(3,873) = 7.94 (10–5)	0.1 < 0.2 (10^–2^)
	0.2	18.13 (3.21)		0.1 < 0.3 (10^–12^)
	0.3	38.19 (3.21)		0.1 < 0.4 (10^–10^);
	0.4	34.94 (3.21)		0.2 < 0.3 (10–5); 0.2 < 0.4 (10^–4^)
Efficiency local	0.1	9.55 (1.28)	*F*(3,873) = 215.07 (10–104)	0.1 > 0.3 (10^–3^)
	0.2	9.70 (1.28)		0.1 > 0.4 (10^–4^)
	0.3	3.68 (1.28)		0.2 > 0.3 (10^–3^)
	0.4	3.47 (1.28)		0.2 > 0.4 (10^–3^)
Eigenvector centrality	0.1	0.98 (0.05)	*F*(3,873) = 382.18 (10–158)	0.1 < 0.2 (10^–3^)
	0.2	1.22 (0.05)		0.1 < 0.3 (10^–24^)
	0.3	1.70 (0.05)		0.1 < 0.4 (10^–94^); 0.2 < 0.3 (10^–11^)
	0.4	2.52 (0.05)		0.2 < 0.4 (10^–72^); 0.3 < 0.4 (10^–34^)
Shortcuts averagerange	0.1	0.48 (0.02)	*F*(3,873) = 123.18 (10–66)	0.1 < 0.2 (10^–25^)
	0.2	0.75 (0.02)		0.1 < 0.3 (10^–84^)
	0.3	1.02 (0.02)		0.1 < 0.4 (10^–148^); 0.2 < 0.3 (10^–25^)
	0.4	1.27 (0.02)		0.2 < 0.4 (10^–80^); 0.3 < 0.4 (10^–22^)
Flow coefficient	0.1	1.49 (0.04)	*F*(3,873) = 115.07 (10–63)	0.1 < 0.2 (10^–3^)
	0.2	1.68 (0.04)		0.1 < 0.3 (10^–23^)
	0.3	2.06 (0.04)		0.1 < 0.4 (10^–59^); 0.2 < 0.3 (10^–11^)
	0.4	2.45 (0.04)		0.2 < 0.4 (10^–41^); 0.3 < 0.4 (10^–12^)
Get components	0.1	0.12 (0.08)	*F*(3,873) = 25.489 (10^–16^)	0.1 < 0.3 (10^–10^)
	0.2	0.15 (0.08)		0.1 < 0.4 (10^–51^)
	0.3	0.82 (0.08)		0.2 < 0.3 (10^–9^)
	0.4	1.87 (0.08)		0.2 < 0.4 (104^–9^); 0.3 < 0.4 (10^–20^)
Topological overlap	0.1	0.34 (0.01)	*F*(3,873) = 501.41 (10–189)	0.1 < 0.2 (10^–34^)
	0.2	0.58 (0.01)		0.1 < 0.3 (10^–110^)
	0.3	0.84 (0.01)		0.1 < 0.4 (10^–177^); 0.2 < 0.3 (10^–35^);
	0.4	1.034 (0.01)		0.2 < 0.4 (10^–94^); 0.3 < 0.4 (10^–23^)
Katz centrality	0.1	219.44 (3.84)	*F*(3,873) = 3.46 (10–2)	0.1 < 0.4 (10^–3^)
	0.2	226.43 (3.84)		
	0.3	229.09 (3.84)		
	0.4	236.10 (3.84)		
k-coreness centrality	0.1	0.35 (0.01)	*F*(3,873) = 409.80 (10–166)	0.1 < 0.2 (10^–21^)
	0.2	0.55 (0.01)		0.1 < S 0.3 (10^–94^)
	0.3	0.83 (0.01)		0.1 < 0.4 (10^–151^); 0.2 < 0.3 (10^–37^)
	0.4	1.01 (0.01)		0.2 < 0.4 (10^–87^); 0.3 < 0.4 (10^–17^)
Matching index	0.1	36.23 (1.59)	*F*(3,873) = 73.80 (10–42)	0.1 < 0.2 (10^–11^)
	0.2	21.02 (1.59)		0.1 < 0.3 (10^–28^)
	0.3	11.26 (1.59)		0.1 < 0.4 (10^–38^)
	0.4	6.40 (1.59)		0.2 < 0.3 (10^–5^); 0.2 > 0.4 (10^–10^)
Modularity finetune	0.1	4.11 (0.10)	NS	
	0.2	4.28 (0.10)		
	0.3	4.37 (0.10)		
	0.4	4.27 (0.10)		
Modularity louvain	0.1	3.53 (0.11)	NS	
	0.2	3.62 (0.11)		
	0.3	3.56 (0.11)		
	0.4	3.68 (0.11)		
Pagerank centrality	0.1	10.05 (0.84)	*F*(3,873) = 8.41 (10–05)	0.1 > 0.3 (10^–3^)
	0.2	10.03 (0.84)		0.1 > 0.4 (10^–3^)
	0.3	6.15 (0.84)		0.2 > 0.3 (10^–3^)
	0.4	5.82 (0.84)		0.2 > 0.4 (10^–3^)
Strength	0.1	1.75 (0.07)	NS	
	0.2	1.60 (0.07)		
	0.3	1.70 (0.07)		
	0.4	1.68 (0.07)		
Subgraph centrality	0.1	7.94 (0.17)	*F*(3,873) = 37.31 (10–22)	0.1 < 0.2 (10^–11^)
	0.2	9.52 (0.17)		0.1 < 0.3 (10^–17^)
	0.3	9.92 (0.17)		0.1 < 0.4 (10^–19^)
	0.4	10.07 (0.17)		
Transitivity	0.1	0.19 (0.003)	*F*(3,873) = 44.96 (10–27)	0.1 > 0.2 (10^–20^)
	0.2	0.15 (0.003)		0.1 > 0.3 (10^–19^)
	0.3	0.15 (0.003)		0.1 > 0.4 (10^–1^)
	0.4	0.18 (0.003)		0.2 < 0.4 (10^–10^); 0.3 < 0.4 (10^–9^)

## Discussion

Anatomical asymmetries, together with image noise and cytoarchitectonic variability, may be responsible for the misplacement of anatomical borders during parcellation, thus affecting network connectivity analyses. The purpose of the present study was to assess network SS, computing VF for several metrics as a measure of their “inertia” to graph structural changes. The use of metrics with a good resilience to small parcellation variations can positively affect the reliability of a functional connectivity analysis. Particularly, the method implemented in this study reproduced what is happening when we try to apply an atlas to a subject’s brain. In this case, each subject will end up with an individual parcellation which will depend on the individual brain structure. We assume that individual brains will be slightly different based on anatomical variability and other individual variables related to the specific acquisition. In this view, each patient carries an “individual error,” depending on his/her/their anatomy as well as on examination-related variables such as motion and differences in acquisition protocol.

The study recreated an unwanted effect and then monitor whether the graph metrics are actually stable enough to infer differences due to diseases. Our analysis was designed to reproduce the individual error and provide a proof of point of its effects on graph metrics. For this purpose, the study individualizes the parcellation to better capture the “individual error burden.” The random simulation also partially and indirectly accounts for the bad alignment between functional data and parcellation. We are in fact stretching and deforming the parcels, and the misregistration behaves in the same way when we think in terms of parcels. Additionally to parcellation errors due to subject variability and to acquisition noise errors, also errors for registration between the individual functional data and parcels propagate with a knock-on effect on brain connectivity analysis. In this context, the simulated variability seen in the graph metrics accounts for a misregistration between the individual functional data and the template space. Using our algorithm, we performed random parcellation variations to reproduce a mean cortical volume variation of the parcels equal to 10%, the minimum coefficient of between-subject variability for a parcellation unit as defined by Kennedy et al. (1998). We also investigated the impact of different datasets (COBRE, IU and ONRC), atlases (DKT and Destrieux), and absolute thresholds (0.1, 0.2, 0.3, and 0.4) on measure generation.

### Spatial Stability

As reported in Figures 3–8, SS results are highly variable among different network metrics. The best SS results were obtained for Characteristic path length, Density, and Transitivity (all VF values below 1%), thus supporting findings reported for these widely used metrics (Salvador et al., 2005; Tomasi and Volkow, 2010; Wee et al., 2014; Prasad et al., 2015; Thomas et al., 2015; Kocevar et al., 2016; Zhao et al., 2016; Zhuo et al., 2017). Moreover, the result of good SS for these measures is further confirmed by recent studies showing excellent test/re-test reliability (TRT) for transitivity and characteristic path length values (Aarabi and Huppert, 2019; Ran et al., 2020; Xiang Y. et al., 2020). Slightly less robust SS was found for several metrics, including modularity (community louvain, modularity finetune and modularity louvain), centrality (closeness, communicability, Eigen-vector, Shortcuts for weighted matrix, Flow coefficient and K-coreness for binary matrix), degree (degree and strength), clustering (Get components and Clustering Coefficient), core (core periphery, assortativity), distance (local Efficiency) and similarity (Topological overlap) measures, as demonstrated by VF values below 10%. Similar results were also obtained in TRT studies, where global graph measures like Clustering coefficient, local Efficiency, and Assortativity revealed fair to good reliability in terms of TRT and intraclass correlation coefficient (ICC) (Ran et al., 2020; Xiang J. et al., 2020). Lower SS resulted for centrality measures, i.e., Katz and Bonacich centrality, which always exhibited VF values higher than 100%. Although Katz centrality measure was firstly introduced for sociometric analysis (Katz, 1953), it has been recently used for different applications in brain connectivity studies, including behaviour characterization of epileptogenic foci (Adkinson et al., 2019) and neuronal activity prediction (Fletcher and Wennekers, 2018). Nevertheless, both Katz and Bonacich centrality metrics can be considered unconventional graph measures, poorly implemented in the clinical field and not yet investigated for reliability assessment. To the best of our knowledge, this is the first study attempting to provide an estimate of the effect of parcellation onto graph theoretical metrics’ values. We evaluated such effect on a wide range of graph metrics (i.e., 24 weighted and 3 binary graph measures). Given the higher reliability of weighted graph metrics compared to their binary counterparts (Xiang J. et al., 2020), this study mainly focused on weighted metrics, excluding binary measures, with the exception of those having a binary definition only (i.e., Subgraph centrality, K-coreness centrality, and Flow coefficient).

### Effect of Atlas

Since reproducibility and reliability investigations are essential for clinical applications of commonly used graph measurements, several efforts were recently made to evaluate how possible changes in acquisition and processing parameters could affect functional connectivity robustness. In this context, the impact of different parcellation atlases (Cao et al., 2014; Arslan et al., 2018; Lacy and Robinson, 2020; Ran et al., 2020), strategies for correlation matrix production (Liang et al., 2012; Telesford et al., 2013), scan length (Birn et al., 2013; Zuo et al., 2013), number of subjects (Termenon et al., 2016), network choice (Braun et al., 2012; Ran et al., 2020), and threshold selection for the adjacency matrix (Lacy and Robinson, 2020; Ran et al., 2020) have been investigated in the literature. Among several methods used to generate parcellation, data-driven approaches showed lower reliability compared to morphology/geometric atlas-based methods, as the subject specificity of the BOLD signal might influence the process of parcellation (Zeng et al., 2019). In order to reduce confounding effects on SS from data-driven approaches, we chose to exploit geometric-based parcellation (standard atlases) for our simulations. Particularly, we used geometric atlases with 2 different numbers of brain parcels (64 and 150 parcels, respectively, for DKT and Destrieux) to assess the effect of graph measures on SS. Significant effects resulted for 74% of the overall network metrics, with centrality-weighted measures (except for closeness centrality), subgraph centrality, degree measures, modularity measures, similarity measures, clustering coefficient, and Get components, showing better SS results for lower granularity, while the opposite trend was found for Assortativity, Characteristic path length, Closeness centrality, Density, Flow coefficient, and Transitivity. The remaining 26% of metrics did not show any significant effect for the atlas choice. Although previous studies investigated an atlas granularity effect on metrics reliability (Cao et al., 2014; Termenon et al., 2016; Zeng et al., 2019; Ran et al., 2020), a definite conclusion cannot be drawn from their results due to heterogeneity. In fact, the mentioned studies relied on very different parameters for metrics estimation (ICC or TRT). For example, Termenon et al. (2016) found high reliability values (in terms of ICC) of local efficiency and clustering for an increasing number of parcels, with global efficiency providing reliable results for coarser parcellations. Among atlas-sensitive metrics, we demonstrated prevalent (70%) SS improvement for a smaller number of parcels, suggesting that higher-granularity parcellations are more prone to misplacing issues. Among the remaining 30% exhibiting the opposite trend, 67% of measures were defined at a global level (i.e., Assortativity, Characteristic path length, Density, and Transitivity). Recently, Ran et al. obtained a trend of better reproducibility (in terms of TRT) of global graph measures (Global efficiency, Clustering coefficient and Betweenness centrality) for finer parcellation (Ran et al., 2020). Conversely, in accordance with our results, a recent study found ICC values related to the functional connectivity correlation matrix to be higher for lower granularity parcellations (Zeng et al., 2019). As far as we observed, SS strongly depends on the network analysis level (i.e., local or global) and this finding is also in line with previous studies on standard reliability. High local fluctuations found in most metrics in the case of finer parcellation might depend on the smaller brain area used to define a node as compared to the percentage of area variation applied in the simulation. Signal-to-noise ratio (SNR) reduction in smaller parcels when compared to larger ones might also be a reason for SS loss, as observed in local metrics. This effect is mitigated for global metrics, since their value reflects a less “node-specific” behaviour of the network. To test the effect of dataset selection, we evaluated datasets with different numbers of subjects and different acquisition parameters.

### Effect of Dataset

Thirty percent of all the metrics did not show any significant effect of dataset on SS. Among the remaining metrics, 79% showed better SS for the larger dataset (COBRE), and 68% displayed worse SS for the smaller dataset (IU). Most of previous studies were performed on a limited number of subjects (Button et al., 2013; Ioannidis, 2014), thus raising the issue that small sample size could be responsible for poorly reliable results. Recent studies demonstrated increasing significance of ICC when a bigger sample size was considered, thus illustrating the role of sample size in reaching statistical significance for reliability assessment (Termenon et al., 2016). Moreover, as scan length is broadly influenced by the number of acquired volumes and the TR of the sequence, both parameters could play a role in reliability improvement for longer scanning times (Andellini et al., 2015).

### Effect of Threshold

Despite the use of binary graphs being attractive as they simplify most of network metrics computation (Garrison et al., 2015, 2016), we decided to focus our analysis on weighted graphs because they demonstrated higher reliability when compared to binary ones (Xiang J. et al., 2020). Absolute thresholds set a minimum value for the correlation coefficient between pairs of nodes, which are considered connected (or not connected) if above (or below) the defined threshold, thus producing a more sparse adjacency matrix for higher thresholds. Despite correlation matrix thresholding being a crucial step for the definition of a network, the optimal method for eliminating non-significant node interactions is still under debate (Simpson et al., 2013). To assess the threshold effect on SS, we computed weighted and binary adjacency matrices by thresholding each correlation matrix over a range of absolute values (0.1, 0.2, 0.3, and 0.4) (Bassett and Bullmore, 2006; Buckner et al., 2009; van den Heuvel and Hulshoff Pol, 2010; Garrison et al., 2015). No significant difference among threshold values (see Table 7) was demonstrated for 11% of the metrics, i.e., Strength, Modularity Finetune, and Louvain. Since few relevant studies have been published on the topic so far, contextualizing these results in the extant literature is difficult. 75% of the metrics significantly affected by threshold variation showed reduced SS at increasing threshold absolute values. This result could be explained considering that increasing threshold values produce highly sparse adjacency matrices, where each change in connection may have a dramatic impact on SS of graph measures. Such finding seems to be in line with previous studies reporting better network reproducibility when adjacency matrices are less sparse (Ran et al., 2020). We can hypothesize this effect being related to the presence of low-correlation values, which are extensively removed when absolute threshold values are increased. Small parcel variations would affect both lower correlations and higher correlations. A similar effect on SS occurs when low correlation values are cut out, since reduction in connections has a strong impact on metrics reliability (Ran et al., 2020). Among the remaining 25%, half of the metrics (Betweenness centrality, Matching index, and Transitivity) did not show a univocal significant trend of SS with increasing threshold absolute values, while the other half (Assortativity, Local Efficiency, and Pagerank) showed increased SS with increasing threshold absolute values. It is not clear why these metrics present such opposite trends, but we can hypothesize that it may be related to the metrics definition; in this context, Pagerank centrality is inversely related to degree (reduced SS at increasing threshold absolute values).

### Limitations

There are some limitations that should be considered for this study. First of all, the number of subjects and datasets included in the analysis and the number of trials were affected by high computational costs, even after the optimization of our algorithm in python. Also, randomly created parcel variations may not exactly reflect parcellation errors as they may occur in real life. To minimize this shortcoming, we performed several random trials. The study also lacks further testing on the effects of acquisition parameters (e.g., sample size, volume number and TR, magnetic field strength, and number of coil channels) on SS. In fact, each included dataset was acquired under different conditions (see Tables 2, 3), limiting possible comparisons. The presented analysis may be considered different from regular pipelines for connectivity analyses. Our study was designed to reproduce individual errors and provide a proof of point of its effects on graph metrics. For this purpose, we individualized the parcellation to every subject to better capture the “individual error burden.” We assumed that individual brains will be slightly different based on anatomical variability and other variables related to the specific exam. Nevertheless, the use of individual parcellations may limit the generalizability of our results to other analyses using the same parcellation for all subjects.

## Conclusion

In conclusion, the proposed method reproduces intersubject parcellation variability to assess the impact of small parcellation changes on global and local connectivity measures. Particularly, the current study identified determinants of network measure variation induced by small parcellation changes. The present study shows that some measures are more prone to larger variations than others. Specifically, Bonacich centrality and Katz centrality have a higher variation factor. Our results showed that SS in terms of VF is affected by threshold choice, since it decreases with increasing threshold for several measures. Moreover, SS seems to depend on atlas choice. Our results suggest to pay close attention to the method the graph structure is built with, as brain parcellation may depend on the implemented method, acquisition noise, registration difficulties, anatomic and physiological characteristics, diseases, age range, and other variables. This variability may produce spatial errors in cerebral cortex parcellation, compromising the reliability of brain connectivity analyses.

## Data Availability Statement

The original contributions presented in the study are included in the article/Supplementary Material, further inquiries can be directed to the corresponding author.

## Author Contributions

FB, AN, and LP made substantial contributions to the conception and design of the work. FB and ML drafted the manuscript. AN, LP, SG, MRi, LF-T, and MR**o** substantially revised the manuscript. FB, ML, AN, LP, and DL contributed to the data interpretation. FB and MM developed the analysis toolbox. FB performed the statistical analysis. All authors contributed to the article and approved the submitted version.

## Conflict of Interest

The authors declare that the research was conducted in the absence of any commercial or financial relationships that could be construed as a potential conflict of interest.

## Publisher’s Note

All claims expressed in this article are solely those of the authors and do not necessarily represent those of their affiliated organizations, or those of the publisher, the editors and the reviewers. Any product that may be evaluated in this article, or claim that may be made by its manufacturer, is not guaranteed or endorsed by the publisher.

## Footnotes

1
http://fcon_1000.projects.nitrc.org/indi/retro/cobre.html

2
http://fcon_1000.projects.nitrc.org/indi/abide/abide_II.html

3
https://bitbucket.org/oriadev/qunex/wiki/UsageDocs/MultiModalQC.md

4
https://surfer.nmr.mgh.harvard.edu/fswiki/QATools

5
https://fsl.fmrib.ox.ac.uk/fsl/fslwiki/MCFLIRT

6
https://pypi.org/project/btc

7
https://networkx.github.io/documentation/stable
