# Supplementary material for: Spatial Stability of Functional Networks: A Measure to Assess the Robustness of Graph-Theoretical Metrics to Spatial Errors Related to Brain Parcellation
Source: Front Neurosci. 2022 Feb 18;15:736524. doi: 10.3389/fnins.2021.736524 (PMC8894326; doi:10.3389/fnins.2021.736524)
Supplement: Supplementary file 1 [file Data_Sheet_1.pdf]

Supplementary Material

1. Graph theoretical measures included in the study

1.1 Degree measures

Degree measures are included in this study.

Degree is the number of edges connected to the node.

Strength is the sum of weights of edges connected to the node (Rubinov and Sporns, 2010).

The assortativity coefficient measures the correlation between nodal degrees on the two opposite sides of a link (Newman, 2002; Leung and Chau, 2007).

1.2 Similarity measures

Similarity measures such as Topological overlap and Matching index were also included in this study. Topological overlap quantifies the similarity of mth step neighbours between a pair of nodes in terms of nodes that are reachable by a path of a maximal length m (Ravasz et al., 2002).

The matching index computes the overlap of connection patterns for any pair of two nodes (Betzel et al., 2013).

1.3 *Physical connectivity measure*

Density is a physical connectivity measure that represents the fraction between actual and possible connections (Sporns, 2006).

1.4 Clustering measures

Three clustering measures were assessed: clustering coefficient, transitivity and Get components. Clustering coefficient is the fraction of triangles around a node, and it is equivalent to the fraction of node’s neighbours that are also each other’s neighbour (Watts and Strogatz, 1998).

Transitivity is an alternative to the clustering coefficient, representing the ratio of triangles to triplets in the network (Newman, 2003).

Get components measure subnetworks in which all pairs of nodes are connected by a path (Corominas-Murtra et al., 2011).

1.5 Community measures

Community structure, modularity and core periphery were computed.
Community structure is a subdivision of the network into nonoverlapping groups of nodes with maximization of within-group edges, and minimization of between-group edges. We computed community with both Louvain detection and finetuning algorithms.
Modularity quantifies how far the network can be subdivided into such groups (Newman, 2006). Modularity was computed with Louvain algorithm only (Newman, 2004, 2006; Newman and Newman, 2010).
Core periphery is a subdivision of the network in two non-overlapping node groups, a core group and a periphery group, with maximization of core group edges, and minimization of periphery group edges (Borgatti and Everett, 2000).

1.6 Distance measures

Distance measures included characteristic path length, global and local efficiency.
Characteristic path length is defined as the average number of edges in the shortest path (minimal sequence of linked nodes) of the network (Watts and Strogatz 1998; Schreiber 2013).
Global efficiency is inversely correlated to the characteristic path length and is defined as the average inverse shortest path length in the network efficiency (Latora and Marchiori, 2001).
The local efficiency equals to global efficiency computed on a node's neighbours (Rubinov and Sporns, 2010).

1.7 Centrality measures

We analysed weighted and binary centrality measures. Weighted centrality measures included Bonacich, Betweenness, eigenvector, shortcuts average range, Pagerank, Closeness, Katz and Communicability. Binary centrality measures were Subgraph, Flow coefficient and k-coreness.
Betweenness centrality is defined as the fraction of all shortest paths in the network that cross a given node (Rubinov and Sporns, 2010).
Eigenvector centrality measures the centrality of a node proportionally to the sum of the centralities of the nodes which it is directly connected to (first degree nodes) (Newman, 2003; Bonacich, 2007).
Katz centrality of a target node is proportional to the cumulative centrality of the nodes which the target is directly connected to, and to the sum of the nodes which it is indirectly connected to (that

connect to the target node through its immediate neighbours). This measure is computed with an attenuation factor α and extra weight provided to immediate neighbours through a parameter β. In our analysis we chose α=0.1 and β=1 (Newman, 2010).

Bonacich centrality is defined as the weighted sum of paths connecting other nodes (Bonacich, 2007).

PageRank centrality is a variant of eigenvector centrality, defined as the stationary distribution of a stochastic process whose states are represented by network nodes. This stationary distribution is achieved by instantiating a Markov chain on a graph.

The PageRank centrality of a node is proportional to the number of steps (or amount of time) spent at that node as a result of such a process (Boldi et al., 2005; Morrison et al., 2005). The process depends on a damping factor $\lambda \in [0\ .\ .\ 1)$, which we set to $\lambda = 0.85$, according to previous literature (Page et al., 1998).

Closeness centrality is defined as the inverse of the average shortest path length from one node to all the other nodes in the network (Freeman, 1978; Rubinov and Sporns, 2010).

Shortcuts identify central edges which cause a significant reduction of characteristic path length in the network (van den Heuvel and Sporns, 2013).

Communicability betweenness centrality identifies nodes that may be regarded as "key players" (having a highly active role). Communicability betweenness centrality of a node depends on possible paths information can pass through, in a way that longer walks carry less importance (Estrada et al., 2009).

The subgraph centrality of a node is the weighted sum of the closed sequences of linked nodes that may visit a single node more than once (walks), with different lengths starting and ending at the node (Zuo et al., 2012).

The flow coefficient is a local centrality measure based on local neighbourhoods, similar to betweenness centrality (Honey et al., 2007).

The k-coreness links the centrality of a node to its k-core, which is the largest subgraph comprising nodes of degree k at least. k-coreness of a node is equal to k if the node belongs to the k-core but does not belong to the (k+1)-core (Hagmann et al., 2008).

Local measures and global measures were calculated for each of the 100 brain networks randomly generated in each subject.

Bibliography

Betzel, R. F., Avena-Koenigsberger, A., Goãi, J., Sporns, O., Griffa, A., Thiran, J. P., et al. (2013). Multi-scale community organization of the human structural connectome and its relationship with resting-state functional connectivity. *Netw. Sci.* 1, 353–373. doi:10.1017/nws.2013.19.

Boldi, P., Santini, M., and Vigna, S. (2005). PageRank : Functional Dependencies. V, 1–23.

Bonacich, P. (2007). Some unique properties of eigenvector centrality. *Soc. Networks*. 29(4), 555-564. doi:10.1016/j.socnet.2007.04.002.

Borgatti, S. P., and Everett, M. G. (2000). Models of corerperiphery structures Stephen. *Soc. Networks* 21, 375–395. Available at: https://www.osapublishing.org/oe/abstract.cfm?uri=oe-19-26-B1.

Corominas-Murtra, B., Rodríguez-Caso, C., Goñi, J., and Solé, R. (2011). Measuring the hierarchy of feedforward networks. *Chaos* 21(1). doi:10.1063/1.3562548.

Estrada, E., Higham, D. J., and Hatano, N. (2009). Communicability betweenness in complex networks. *Phys. A Stat. Mech. its Appl.* 388, 764–774. doi:10.1016/j.physa.2008.11.011.

Falk Schreiber (2013). Characteristic Path Length. *DUBITZKY, Werner, ed., Olaf WOLKENHAUER, ed., Kwang-Hyun CHO, ed., Hiroki YOKOTA, ed.. Encycl. Syst. Biol.*, NY:Springer, pp. 395. ISBN 978-1-4419-9862–0. doi:10.1007/978-1-4419-9863-7_1460.

Freeman, L. C. (1978). Centrality in social networks conceptual clarification. *Soc. Networks*. 1(3), 215-239. doi:10.1016/0378-8733(78)90021-7.

Hagmann, P., Cammoun, L., Gigandet, X., Meuli, R., Honey, C. J., Van Wedeen, J., et al. (2008). Mapping the structural core of human cerebral cortex. *PLoS Biol.* 6, 1479–1493. doi:10.1371/journal.pbio.0060159.

Honey, C. J., Kötter, R., Breakspear, M., and Sporns, O. (2007). Network structure of cerebral cortex shapes functional connectivity on multiple time scales. *Proc. Natl. Acad. Sci. U. S. A.* 104, 10240–10245. doi:10.1073/pnas.0701519104.

Latora, V., and Marchiori, M. (2001). Efficient behavior of small-world networks. *Phys. Rev. Lett.* 87(19). doi:10.1103/PhysRevLett.87.198701.

Leung, C. C., and Chau, H. F. (2007). Weighted assortative and disassortative networks model. *Phys. A Stat. Mech. its Appl.* 378, 591–602. doi:10.1016/j.physa.2006.12.022.

Morrison, J. L., Breitling, R., Higham, D. J., and Gilbert, D. R. (2005). GeneRank: Using search engine technology for the analysis of microarray experiments. *BMC Bioinformatics* 6, 1–14. doi:10.1186/1471-2105-6-233.

Newman, M. (2010). *Networks: An Introduction*. doi:10.1093/acprof:oso/9780199206650.001.0001.

Newman, M. E. J. (2002). Assortative Mixing in Networks. *Phys. Rev. Lett.* 89, 1–5.

doi:10.1103/PhysRevLett.89.208701.

Newman, M. E. J. (2003). The structure and function of complex networks. *SIAM Rev*. 45(2),167-256. doi:10.1137/S003614450342480.

Newman, M. E. J. (2004). Fast algorithm for detecting community structure in networks. *Phys. Rev. E - Stat. Physics, Plasmas, Fluids, Relat. Interdiscip. Top.* 96(6). doi:10.1103/PhysRevE.69.066133.

Newman, M. E. J. (2006). Modularity and community structure in networks. *Proc. Natl. Acad. Sci.* 103(23), 8577–8582. *U. S. A.* doi:10.1073/pnas.0601602103.

Newman, M., and Newman, M. E. J. (2010). Mathematics of networks. *Networks*, 109–167. doi:10.1093/acprof:oso/9780199206650.003.0006.

Page, L., Brin, S., Motwani, R., and Winograd, T. (1998). The PageRank Citation Ranking: Bringing Order to the Web. *World Wide Web Internet Web Inf. Syst.* doi:10.1.1.31.1768.

Ravasz, E., Somera, A. L., Mongru, D. A., Oltvai, Z. N., and Barabási, A. L. (2002). Hierarchical organization of modularity in metabolic networks. *Science*, 297(5586), 1551–1555. doi:10.1126/science.1073374.

Rubinov, M., and Sporns, O. (2010). Complex network measures of brain connectivity: Uses and interpretations. *Neuroimage* 52, 1059–1069. doi:10.1016/j.neuroimage.2009.10.003.

Sporns, O. (2006). Small-world connectivity, motif composition, and complexity of fractal neuronal connections. *BioSystems* 85, 55–64. doi:10.1016/j.biosystems.2006.02.008.

van den Heuvel, M. P., and Sporns, O. (2013). Network hubs in the human brain. *Trends Cogn. Sci.* 17(12),683-696. doi:10.1016/j.tics.2013.09.012.

Watts, D. J., and Strogatz, S. H. (1998). Strogatz - small world network Nature. *Nature* 393, 440–442. Available at: https://www.ncbi.nlm.nih.gov/pubmed/9623998.

Zuo, X. N., Ehmke, R., Mennes, M., Imperati, D., Castellanos, F. X., Sporns, O., et al. (2012). Network centrality in the human functional connectome. *Cereb. Cortex* 22, 1862–1875. doi:10.1093/cercor/bhr269.
